# A systematic review and meta-analysis of the impact of clopidogrel responsiveness on ischemic and bleeding complications after noncoronary endovascular procedures

**DOI:** 10.1016/j.jvs.2025.10.101

**Published:** 2025-12-15

**Authors:** Ashling L. Zhang, Caroline E. Crone, Georges Jreij, Aidan P. Wiley, Abbey N. Loy, Joy Y. Chai, Sarasijhaa K. Desikan, John D. Sorkin, Ann H. Kim, Brajesh K. Lal

**Affiliations:** a Department of Surgery, University of Maryland School of Medicine, *Baltimore, MD*; b Center for Vascular Research, University of Maryland School of Medicine, *Baltimore, MD*; c Pharmacogenomics, Baltimore Veterans Affairs Medical Center, *Baltimore, MD*; d Department of Vascular Surgery, University of Maryland School of Medicine, *Baltimore, MD*; e Vascular Surgery, Baltimore Veterans Affairs Medical Center, *Baltimore, MD*; f Department of Medicine, University of Maryland School of Medicine, *Baltimore, MD*; g Geriatrics Research, Education, and Clinical Center, Baltimore Veterans Affairs Medical Center, *Baltimore, MD*

**Keywords:** Peripheral artery disease, Lower extremity intervention, Neuroendovascular intervention, Clopidogrel, Genotyping, Platelet function testing

## Abstract

**Objective:**

Short- or long-term periprocedural clopidogrel, with or without aspirin, is standard of care after several endovascular interventions. However, clopidogrel fails to prevent platelet aggregation in 15% to 50% of the population due to mutations in the *CYP2C19* gene. Two methodologies, *CYP2C19* genotyping and platelet function testing (PFT), predict clopidogrel response, but their utility in preventing complications after endovascular interventions is underappreciated. This meta-analysis (1) characterizes current literature on genotyping and PFT as means of identifying clopidogrel response and (2) evaluates the impact of abnormal clopidogrel response on postprocedural ischemic complications (stent thrombosis, restenosis, reintervention, amputation, revascularization, transient ischemic attack/stroke, or myocardial infarction) and bleeding complications.

**Methods:**

We conducted a systematic review and meta-analysis of papers reporting *CYP2C19* genotyping or PFT to assess clopidogrel response in patients undergoing noncoronary endovascular interventions. A total of 272 papers were screened. After excluding 263 papers, nine articles remained. We described the distribution of poor responders obtained using genotyping and PFT. We compared the odds of developing ischemic complications in poor vs normal clopidogrel responders. We compared the odds of developing bleeding complications in hyper-responders vs normal clopidogrel responders.

**Results:**

Four papers assessing clopidogrel response by genotyping and three by PFT reported on ischemic complications. The mean prevalence of poor response was 43.2% ± 10.1% by genotyping and 23.2% ± 8.9% by PFT. All patients received postprocedural clopidogrel. Cumulative ischemic complication rates were 35.1% for poor responders and 14.0% for normal responders. Higher odds of ischemic complications were observed in poor clopidogrel responders identified by genotyping (odds ratio [OR]: 2.8, 95% confidence interval [CI]: 2.1-3.8, *P* < .001) or by PFT (OR: 6.3, 95% CI: 2.0-20.0, *P* = .02). Three papers assessed bleeding complications in patients undergoing neuroendovascular interventions. There was no difference in the odds of major bleeding events between hyper-responders and normal responders (OR: 6.2, 95% CI: 0.6-61.7, *P* = .12). The low number of papers precluded a formal comparison of effectiveness of genotyping vs PFT in predicting complications.

**Conclusions:**

Among patients receiving clopidogrel after endovascular interventions, poor responders experience ischemic complications more frequently. Genotyping chips and PFT assay kits are commercially available and effectively detect poor clopidogrel responders. Genotyping has the advantage of detection before clopidogrel initiation. Rigorous studies are needed to establish the preferred testing modality and to determine whether test result-driven modification of antiplatelet therapy reduces periprocedural complications. (J Vasc Surg 2026;83:1249-58.)

Periprocedural antiplatelet therapy is standard of care for endovascular interventions. Most often delivered as dual-antiplatelet therapy (DAPT), it typically includes aspirin and an inhibitor of P2Y12—a chemoreceptor involved in platelet aggregation—of which clopidogrel is most frequently prescribed.^1,2^ DAPT is recommended after percutaneous coronary intervention (PCI) to prevent ischemic complications such as myocardial infarction (MI), transient ischemic attack (TIA), stroke, or stent thrombosis.^3–8^ This recommendation has historically been extrapolated to patients undergoing noncoronary endovascular interventions.^9^ The 2024 American Heart Association guideline makes a moderate recommendation (limited evidence) for DAPT with a P2Y12 antagonist and aspirin after lower-extremity revascularization for peripheral artery disease (PAD).^9–12^ The European Society for Vascular Surgery makes similar recommendations for patients with low bleeding risk.^13^ As a result, many, if not most surgeons, prescribe short-term DAPT with aspirin and clopidogrel, or clopidogrel alone, after an endovascular intervention.^12,14–16^

*CYP2C19* is the primary cytochrome p450 enzyme responsible for metabolizing clopidogrel to its bioactive form, which inhibits platelet activity by blocking activation P2Y12 receptor activation (Fig 1).^17,19–22^ There are over 50 single nucleotide polymorphisms in the *CYP2C19* gene.^17^ The wild-type CYP2C19*1 allele imparts normal function and occurs in 44.5% to 60.5% of the population. CYP2C19*2 and CYP2C19*3 cause loss of function (LOF) of the enzyme.^17^ Individuals can also carry the gain of function (GOF) allele, CYP2C19*17. Allele frequencies vary by ethnicity; LOF alleles occur more frequently in East Asian (37.7%) and South Asian (34.4%) populations, and GOF alleles occur more frequently in populations of African (23.5%) and European (22.4%) descent (Supplementary Table I, online only). Knowing a patient’s *CYP2C19* genotype helps anticipate their responsiveness to clopidogrel (Table I).^22^ Clopidogrel responsiveness can also be measured by platelet function testing (PFT), which assesses platelet aggregation using light transmission platelet aggregometry or impedance whole blood aggregometry.^23,24^ Ultimately, 15% to 50% of people do not properly metabolize clopidogrel, suggesting a strong population-level impact of these common *CYP2C19* alleles.^25^ Poor clopidogrel response can lead to uninhibited platelet aggregation, which could increase ischemic complications. It is therefore necessary to identify which patients may benefit from alternative antiplatelet therapy to minimize ischemic complications and optimize therapeutic outcomes. We performed a systematic review and meta-analysis to better understand the impact of clopidogrel response on the rate of ischemic complications and bleeding complications after noncoronary endovascular interventions. We also characterized existing literature on genotyping and PFT as methods of assessing clopidogrel response.

## METHODS

### Literature search.

We conducted our review according to the Preferred Reporting Items for Systematic reviews and Meta-Analyses (PRISMA).^26^ On September 12, 2024, we screened the PubMed, Embase, and Cochrane databases for the search terms “clopidogrel” AND “endovascular” AND (“genotype” OR “platelet-function testing”). We uploaded all papers to Covidence and eliminated duplicates. C.E.C. and G.J. screened all abstracts and reviewed relevant papers in full. B.K.L. provided a third-party review for conflicting decisions.

### Inclusion and exclusion criteria.

We included papers reporting ischemic and/or bleeding complications after noncoronary endovascular interventions in patients receiving periprocedural clopidogrel (n = 30). We excluded papers that reported on open vascular surgical procedures (n = 3), did not characterize patients as normal or abnormal responders to clopidogrel by *CYP2C19* genotyping or PFT (n = 3), or did not report postintervention ischemic or bleeding complication rates in these two groups separately (n = 1). We excluded papers where the full text was unavailable (n = 2) and any reviews, conference proceedings, or editorials (n = 12).

### Definitions, data extraction, and outcomes.

Poor clopidogrel response was determined by either genotyping or PFT results. With genotyping, we defined poor clopidogrel responders as patients with at least one LOF allele and hyper-responders as patients with at least one GOF allele. With PFT, different techniques and thresholds for interpreting results were used to define poor, normal, and hyper-responders across papers.

C.E.C., G.J., and A.L.Z. extracted the title, authors, year of publication, number of subjects, and details of the patient population (including procedure type and indication for the procedure) from each paper. We extracted the number of poor, normal, or hyper-responders to clopidogrel and whether this was determined by genotyping and/or PFT. If PFT was used, we also extracted the specific test used and thresholds for determining poor, normal, or hyper-response.

The primary outcome of our meta-analysis was ischemic complications. Because of variation in the definition of ischemic complications between papers, this was defined as a composite of stent thrombosis, restenosis, reintervention, amputation, TIA/stroke, or MI. We extracted the number of poor vs normal responders experiencing an ischemic complication from each paper. We assessed a secondary outcome, bleeding complications, by extracting the number of hyper- vs normal responders who experienced bleeding complications.

### Data analysis.

We conducted three pairwise metaanalyses. A pairwise meta-analysis compares data from two different intervention groups to determine a pooled estimate of effect.^27^ All patients were on periprocedural clopidogrel. The first meta-analysis examined the odds of an ischemic complication among poor vs normal responders classified by *CYP2C19* genotype (n = 4). The second meta-analysis examined the odds of an ischemic complication among poor vs normal responders classified by PFT (n = 3). The third meta-analysis examined the odds of bleeding complications among hyper- vs normal responders (n = 3). If the metric of heterogeneity, I^2^, was ≥50%, we performed a random-effects meta-analysis. Otherwise, we performed a fixed-effects meta-analysis. A two-tailed *P* value <.05 was considered significant. We used R software for all calculations and Forest plots (version 4.3.2; R Foundation for Statistical Computing).

## RESULTS

**Fig 2.**
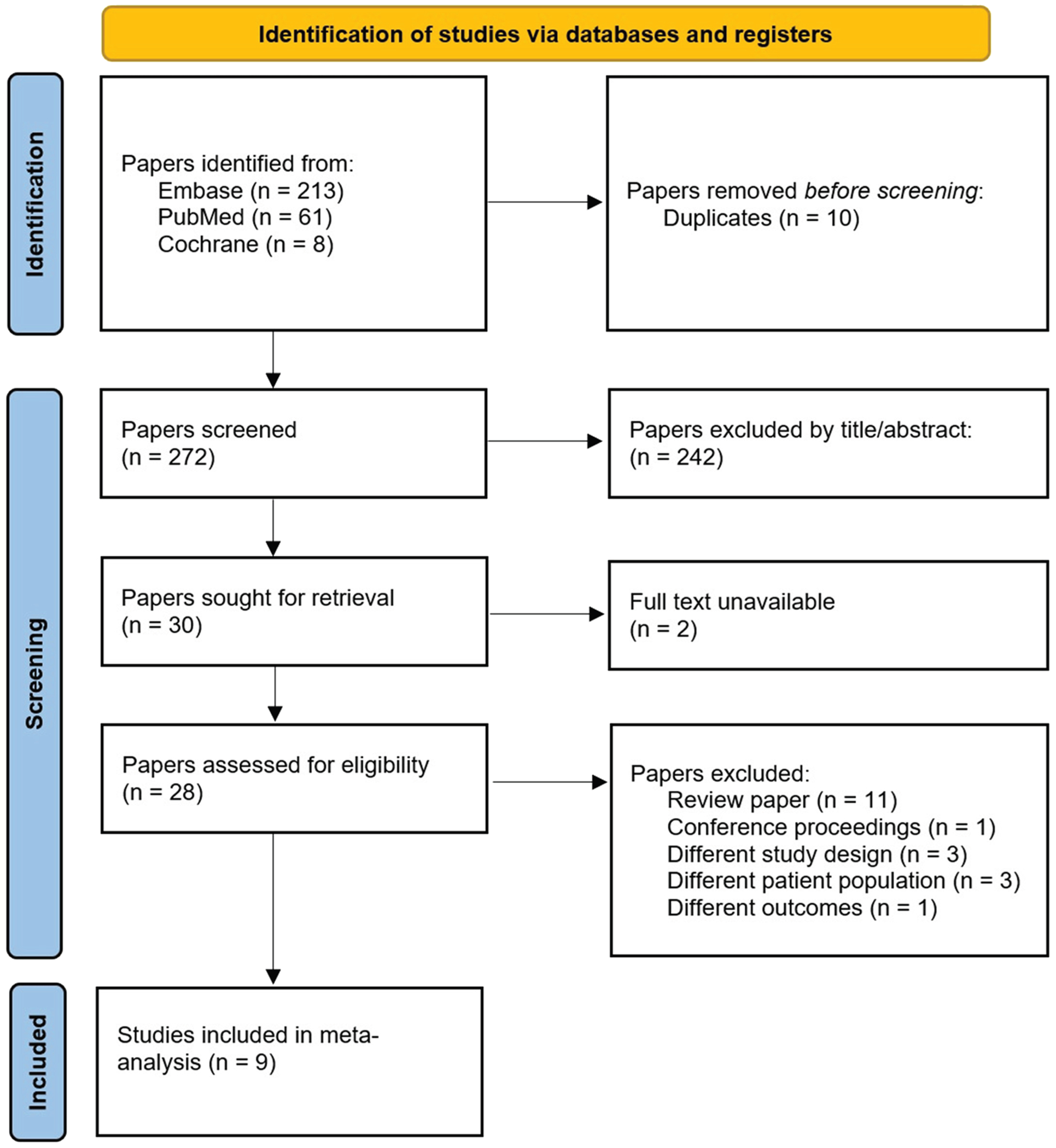
PRISMA diagram of included and excluded papers. Systematic review conducted in Covidence. Template for the PRISMA diagram from Page et al.^26^ PRISMA, Preferred Reporting Items for Systematic Reviews and Meta-Analyses.

In total, 282 papers were identified (Fig 2). After excluding duplicates, 272 were screened for inclusion. Ultimately, nine papers fulfilled all inclusion and exclusion criteria and were included in the meta-analysis.^28–36^ None of the papers were randomized clinical trials.

### Ischemic complications based on clopidogrel response determined by genotype.

Four papers evaluated ischemic complications after endovascular interventions in patients on periprocedural clopidogrel stratified by *CYP2C19* genotype (Supplementary Table II, online only).^28–31^ They varied in the details of the patient population, timing of clopidogrel therapy relative to intervention, and definition of an ischemic complication. Three papers focused on endovascular procedures for lower extremity PAD. Guo et al^29^ studied 50 patients receiving at least 5 days of preintervention and 1 year of postintervention DAPT (aspirin and clopidogrel) for superficial femoral artery occlusive disease. They reported increased ischemic complications (composite of restenosis or occlusion) (57.7% vs 16.7%, *P* = .004) and reduced primary patency (73.1% vs 34.6%, *P* = .006) within 1 year of intervention among patients with at least one *CYP2C19* LOF allele compared with those without any LOF alleles. Lee et al^30^ revascularized 278 patients with critical limb ischemia and maintained them on clopidogrel alone for 1 year. Having at least one *CYP2C19* LOF allele was associated with a greater risk of amputation within 1 year of intervention (hazard ratio: 2.2, 95% confidence interval [CI]: 2.0-2.5, *P* = .01). Chang et al^28^ undertook a prospective cohort study examining major adverse limb events, defined as amputation or reintervention during the study period, in patients with critical limb ischemia on clopidogrel (alone or combined with other antiplatelet therapy) postintervention. They found that major adverse limb events occurred more frequently in patients with one or more *CYP2C19* LOF alleles vs those with no LOF alleles (41.3% vs 18.8%) but did not determine statistical significance. Lin et al^31^ reported a prospective cohort of 108 patients undergoing neuroendovascular procedures with at least 3 days of preintervention DAPT (aspirin and clopidogrel). They found no difference in TIA or ischemic strokes within 3 months of the intervention across all genotypes. Interestingly, patients with at least one *CYP2C19* GOF allele demonstrated a greater frequency of ischemic complications than those with no GOF or LOF alleles (32.1% vs 11.4%, *P* = .04).

**Fig 3.**
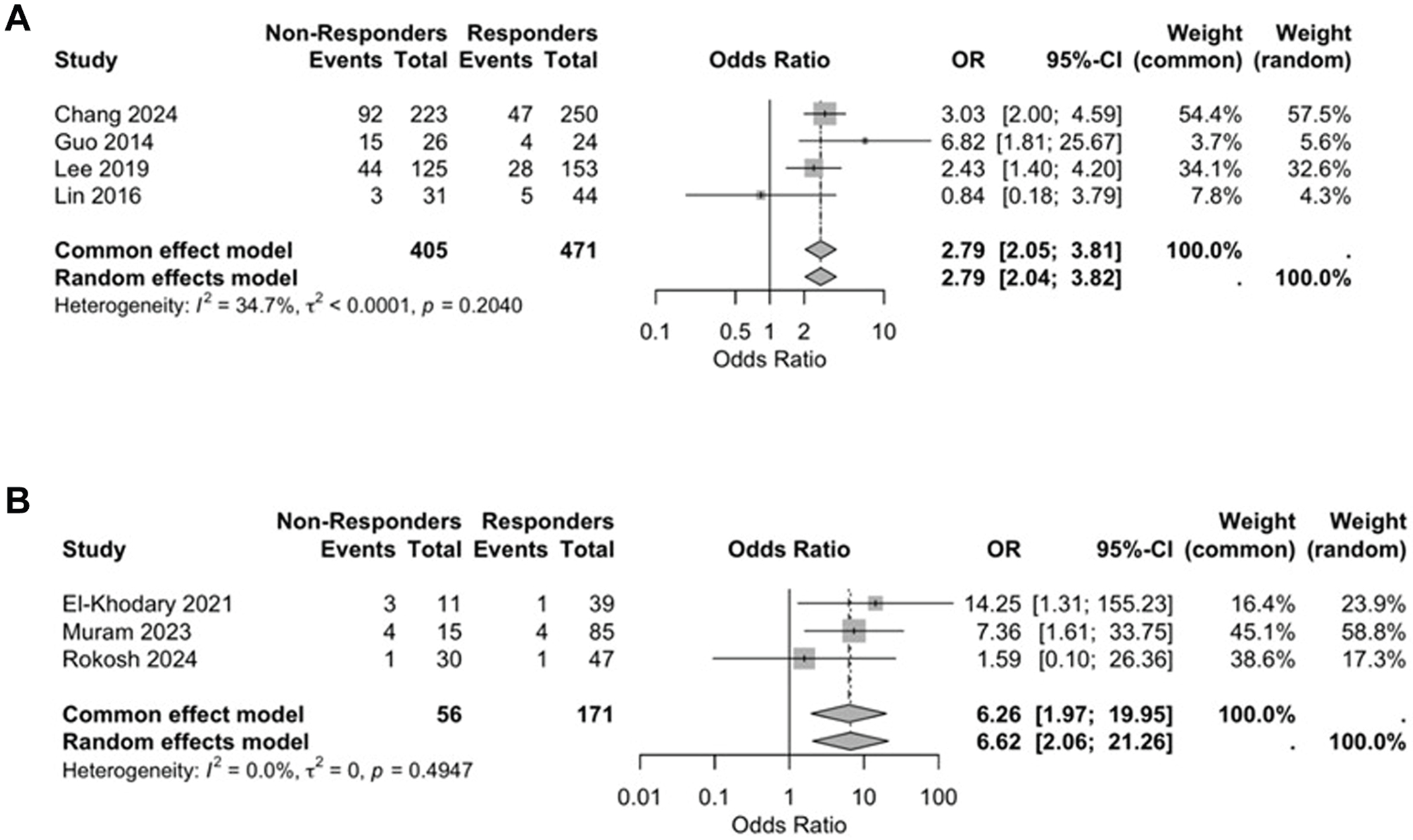
Forest plot of odds ratios for ischemic complications in poor responders vs responders identified by **(A)** genotyping for *CYP2C19* or **(B)** platelet function testing. **A,** A Forest plot of odds ratios for adverse ischemic complications in poor responders vs responders identified by genotyping for *CYP2C19.* When limited to papers evaluating lower extremity endovascular interventions only,^28–30^ the common-effects model produces an odds ratio of 3.0, 95% CI: 2.2-4.1, and *P* < .001. **B,** A Forest plot of odds ratios for adverse ischemic events in poor responders vs responders identified by platelet function testing. When limited to papers evaluating neuroendovascular interventions only,^33,34^ the common-effects model produces an odds ratio of 4.7, 95% CI: 1.2-18.2, and *P* = .02. *CI*, Confidence interval; *OR*, odds ratio.

The pooled meta-analysis of the four papers included 405 poor clopidogrel responders and 471 normal responders based on *CYP2C19* genotype (Fig 3, A). Across both PAD and neuroendovascular procedures, poor responders had 2.8 times higher odds of experiencing ischemic complications compared with normal responders (odds ratio [OR]: 2.8, 95% CI: 2.1-3.8, *P* < .001). When restricted to the three papers in patients with PAD, poor responders were still more likely to experience ischemic complications (OR: 3.0, 95% CI: 2.2-4.1, *P* < .001). A separate meta-analysis was not conducted for neuroendovascular procedures alone, as only one paper described this patient population.

### Ischemic complications based on clopidogrel response determined by platelet function testing.

Three papers evaluated ischemic complications based on clopidogrel response determined by PFT (Supplementary Table III, online only).^32–34^ The papers varied by patient population, type of test, threshold for defining poor clopidogrel response, timing of clopidogrel therapy relative to intervention, and definition of ischemic complications. El-Khodary et al^32^ conducted a prospective cohort study of PAD revascularization procedures in 50 patients on 6 months of postintervention clopidogrel. Poor clopidogrel responders were defined as having platelet aggregation ≥60% on a platelet aggregation assay. Poor responders were more likely to experience major amputation (27.3% vs 3.6%, *P* = .03) and death within 1 year of intervention (27.3% vs 3.6%, *P* = .03) than normal responders. There was no significant difference in frequency of reintervention (18.2% vs 3.6%, *P* = .12), nonfatal MI (9.1% vs 3.6%, *P* = .40), or stroke (18.2% vs 3.6% *P* = .12). Muram et al^33^ and Rokosh et al^34^ focused on carotid artery stenting. Muram et al^33^ defined poor clopidogrel responders as impedance >5 Ω on whole-blood impedance aggregometry. Their prospective cohort of 100 patients received DAPT (aspirin and clopidogrel) at least 7 days before intervention and 3 months after intervention. Poor responders experienced more ischemic strokes within 90 days of intervention (26.7% vs 4.7%, *P* = .003). Rokosh et al^34^ defined poor clopidogrel responders as ≥194 P2Y12 reaction units on the VerifyNow P2Y12 assay. Their retrospective cohort comprised 92 patients receiving at least 7 days of clopidogrel before intervention. No significant differences were identified in ischemic strokes (3.3% vs 0.0%, *P* = .35) or MIs (0% vs 2.1%, *P* = 1.0) between poor and normal responders.

The pooled meta-analysis of the above three papers included 56 poor clopidogrel responders and 171 normal responders based on PFTs (Fig 3, B). Poor responders had 6.3 times higher odds of experiencing ischemic complications compared with normal responders (OR: 6.3, 95% CI: 2.0-20.0, *P* = .002). When restricted to the two papers focusing on neuroendovascular procedures (specifically carotid artery stenting), poor responders had higher odds of experiencing an ischemic complication than normal responders (OR: 4.7, 95% CI: 1.2-18.2, *P* = .02). A separate meta-analysis was not conducted for PAD endovascular procedures alone, as only one paper described this patient population.

### Bleeding complications in clopidogrel hyper-responders.

Three papers evaluated bleeding complications after neuroendovascular intervention in clopidogrel hyper- vs normal responders (Supplementary Table IV, online only).^31,35,36^ The papers varied in the methods of identifying hyper-responders, timing of bleeding, and definition of bleeding. Lin et al^31^ included 28 clopidogrel hyper-responders based on the presence of at least one CYP2C19*17 GOF allele. There was no difference in intra- or extracranial bleeding within 3 months after intervention across all studied genotype groups. Goh et al^36^ and Kashiwazaki et al^35^ sought to identify an optimal threshold for defining clopidogrel hyper-response using PFT. Goh et al^36^ prospectively studied 47 patients receiving 3 days of DAPT (aspirin and clopidogrel) before intervention and found that the median P2Y12 percent inhibition was higher among patients with major bleeding vs those without (94% vs 24%, *P* = .008). They further reported that ≥72% P2Y12 percent inhibition on PFT was the optimal cutoff for defining clopidogrel hyper-responders. Major bleeding was defined as intracranial hemorrhage, retroperitoneal hematoma, large groin hematoma, or bleeding requiring additional intervention. Kashiwazaki et al^35^ used a threshold of ≥74% P2Y12 percent inhibition to define clopidogrel hyper-responders in their study of 66 patients receiving 14 days of DAPT (aspirin and clopidogrel) before intervention. Hyper-responders had more major bleeding events within 30 days of intervention (40.0% vs 6.3%, *P* = .001).

**Fig 4.**
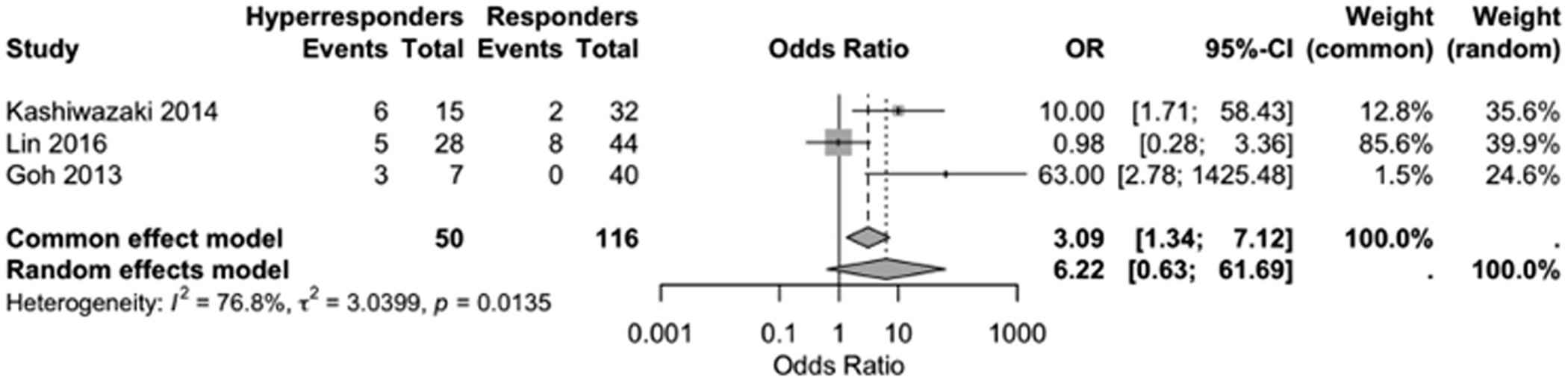
Forest plot of odds ratios for bleeding complications in hyper-responders vs responders identified by genotyping or platelet function testing. When limited to platelet function testing only,^35,36^ the common-effects model produces an odds ratio of 15.7, 95% CI: 3.5-69.9, and *P* < .001. The random-effects model produces an odds ratio of 15.7, 95% CI: 3.3-74.3, and *P* < .001. *CI*, Confidence interval; *OR*, odds ratio.

The pooled meta-analysis of the above three papers included 50 hyper-responders and 116 normal responders (Fig 4). Among patients undergoing neuroendovascular interventions, there was no difference in the odds of major bleeding events between hyper- and normal responders (OR: 6.2, 95% CI: 0.6-61.7, *P* = .12). When restricted to the two papers that used PFT, hyper-responders were more likely to experience major bleeding than normal responders (OR: 15.7, 95% CI: 3.5-69.9, *P* < .001). A separate meta-analysis was not conducted for hyper-responders identified by genotyping, as only one study used this method.

## DISCUSSION

Our systematic review and meta-analysis shows that poor responders to clopidogrel are at a higher risk of incurring ischemic complications when administered clopidogrel (with or without aspirin) for endovascular interventions compared with normal responders, both when clopidogrel response is determined by genotype (OR: 2.8, 95% CI: 2.1-3.8, *P* < .001) and by PFT (OR: 6.3, 95% CI: 2.0-20.0, *P* = .002). Ischemic complications were reported as a composite of stent thrombosis, restenosis, amputation, revascularization, TIA/stroke, or MI. The increased risk remains significant regardless of the type of testing, *CYP2C19* genotyping or PFT phenotyping, used to determine clopidogrel response. Data on bleeding risk in clopidogrel hyper-responders undergoing endovascular interventions are less conclusive and require further evaluation.

Our review shows that investigations on the clinical impact of clopidogrel responsiveness on noncoronary endovascular interventions are limited. On initial screening, the vast majority of papers were excluded for studying coronary endovascular interventions. Ultimately, only nine papers enrolling a total of 1264 patients addressed our question. After endovascular procedures, poor clopidogrel responders identified by genotyping had higher odds of experiencing ischemic complications compared with normal responders (OR: 2.8, 95% CI: 2.1-3.8, *P* < .001). Poor responders identified by PFTs also had higher odds of ischemic complications compared with normal responders (OR: 6.3, 95% CI: 2.0-20.0, *P* = .002). Further review of specific patient populations showed that genotypic determination of poor clopidogrel response in patients undergoing PAD interventions and PFT determination of poor clopidogrel response in patients undergoing neuroendovascular interventions are associated with higher risk of ischemic complications than normal response. Evidence using PFT in PAD interventions or genotyping in neuroendovascular interventions was limited to one study each. Mutations in the *CYP2C19* gene are known to lead to loss of clopidogrel responsiveness.^37,38^ It was only recently that studies in patients undergoing PCI investigated the association between *CYP2C19* genotypes and postintervention cardiovascular events.^39,40^ They demonstrated an increased risk of adverse cardiovascular events, including stent thrombosis and MI, among LOF allele carriers. One systematic review and meta-analysis included 32 studies involving 42,016 patients and found that carriers of LOF alleles (*2, *3) exhibited reduced platelet inhibition and an increased risk of cardiovascular events after PCI (relative risk [RR]: 1.2; 95% CI: 1.1-1.3).^38^

Our results suggest that although identification of clopidogrel hyper-responders by PFT may predict postintervention bleeding after neuroendovascular interventions, the role for genotypic identification of hyper-responders remains undetermined. In fact, the one study that identified hyper-responders using genotyping (CYP2C19*17 alleles) in such patients found an increased risk of ischemic complications, which contradicts the hypothesis that hyper-response is associated with fewer ischemic complications and more bleeding. There is a lack of data on bleeding events in genotyping-identified hyper-responders after PAD interventions. Therefore, based on current literature, there is insufficient evidence to support the adjustment of antiplatelet regimens based on *CYP2C19* genotype to prevent bleeding. Ticagrelor and prasugrel have been associated with increased bleeding rates compared with clopidogrel and may therefore not be appropriate alternatives in clopidogrel hyper-responders.^41–45^

Although our results suggest that both genotyping and PFT can predict risk of postintervention ischemic complications, there are insufficient data to determine which is the better method to identify poor responders to clopidogrel. *CYP2C19* genotyping predicts clopidogrel response before any medication is administered, allowing for early modification of antiplatelet therapy.^20^ PFT provides a more comprehensive understanding of clopidogrel response, as it encompasses all the factors that may impact phenotype, such as the genotype, drug interactions, compliance, and comorbidities.^42,46^ However, because PFT can only be performed after starting clopidogrel and therefore exposes patients to potential ischemic complications, genotyping may have greater utility in providing preintervention data to guide postintervention antiplatelet therapy.^20^ The feasibility of each test varies across medical facilities due to differences in cost, availability, and laboratory logistics. Genotyping and PFT have been compared in patients undergoing coronary endovascular intervention, but no studies have directly compared their ability to predict complications after PAD and neuroendovascular interventions.

“Tailored,” “targeted,” or “guided therapy” refers to the selection of postintervention antiplatelet therapy directed by *CYP2C19* genotype or PFT results. Ticagrelor and prasugrel are P2Y12 inhibitors that are not metabolized by *CYP2C19*, making them attractive alternatives to clopidogrel in *CYP2C19* LOF carriers. They are not routinely used in place of clopidogrel because they carry a greater risk of bleeding.^37,41,47^ During our literature review, we encountered four papers investigating the impact of targeted therapy.^48–51^ The studies used a variety of medications to substitute for clopidogrel in patients identified to be poor clopidogrel responders (eg, ticagrelor,^48^ cilostazol,^50^ and adjusted dose of clopidogrel^51^). Most studies were not designed to detect statistically significant differences in clinical outcomes between poor responders on clopidogrel vs poor responders on tailored therapy. The one study of tailored therapy after endovascular intervention for PAD only monitored complications among recipients of tailored therapy and did not include a control group for comparison.^49^ None of the studies found a statistically significant difference in ischemic or bleeding complications between the two groups of patients. Furthermore, the studies were limited by sample size and number of events; the largest number of ischemic complications in a single study was 21.^50^ The variation in study design precludes definitive conclusions on potential benefit of tailored therapy in patients undergoing PAD or neuroendovascular procedures. The clinical benefit of genotype-guided tailored antiplatelet therapy after PCI was evaluated in *TAILOR-PCI*, which compared standard therapy with genotype-guided selection of antiplatelet agents.^18^ Although the primary end point did not reach statistical significance, trends favored genotype-guided escalation to prasugrel or ticagrelor in LOF carriers. Guidelines from organizations such as the Clinical Pharmacogenetics Implementation Consortium support genotype-guided therapy in specific clinical scenarios.^52^ Meta-analyses, such as the one by Zhang et al,^53^ reinforced these findings. The study demonstrated that ticagrelor and prasugrel significantly reduced ischemic complications after PCI compared with clopidogrel in *CYP2C19* loss-of-function carriers (RR: 0.7; 95% CI: 0.60.8), but not in noncarriers (RR: 1.0; 95% CI: 0.8-1.3).

We have identified gaps in knowledge regarding clopidogrel responsiveness and noncoronary endovascular interventions that must be addressed before recommendations can be safely made. Clinical studies directly comparing genotyping vs PFT for predicting postintervention complications are necessary to select the optimal method of identifying poor responders. Randomized controlled trials are needed to determine whether tailoring antiplatelet therapy to a patient’s clopidogrel response profile reduces ischemic complications after noncoronary endovascular procedures.^54,55^ Such trials should obtain clopidogrel responsiveness data preintervention, provide a standard alternative to clopidogrel for poor responders postintervention, identify patient and/or procedural characteristics that predict who would benefit most from tailored therapy, and follow patients past the intended duration of antiplatelet therapy to identify long-term outcomes. Additional data showing that clopidogrel hyper-response increases postintervention bleeding complications would be needed before considering similar trials in hyper-responders. Once the clinical impact is quantified, cost-effectiveness evaluations can be undertaken to further inform whether clopidogrel response-guided antiplatelet therapy should be adopted in clinical practice. Finally, additional studies showing that clopidogrel hyper-response increases postintervention bleeding complications would be needed before considering similar trials in hyper-responders.

Our study is inherently limited by publication bias, whereby papers that are published and available for inclusion in our meta-analysis are more likely to report statistically significant findings. This effect may falsely inflate the significance of our results. This metaanalysis was also limited by the small number of qualifying papers. Further, the included papers demonstrated heterogeneity in sample size, type of intervention, definition of poor clopidogrel response, and definition of adverse outcomes. Such variability limits our ability to generalize our findings to a wider population and draw conclusions regarding the specific conditions under which clopidogrel response is associated with clinical outcomes. In light of these limitations, the need for further studies using standardized methods to identify clopidogrel response and outcomes becomes even more apparent.

## CONCLUSIONS

Both genotyping and PFT assess patient clopidogrel responsiveness, and current studies demonstrate an association with ischemic complications. According to one recent systematic review focused on noncoronary interventions, the literature indicates that poor clopidogrel response is associated with ischemic complications.^56^ Our meta-analysis now brings statistical significance to this finding. Genotyping provides a onetime assessment of metabolic capacity, whereas PFT captures the net effect of multiple influences, including drug interactions and adherence. Evidence is insufficient to support that clopidogrel hyper-response is associated with postintervention bleeding complications, except in the specific case of hyper-responders identified by PFT who undergo neuroendovascular procedures. Collectively, current information indicates higher rates of ischemic complications in patients who are nonresponders to clopidogrel. Level 1 data do not exist on a clinical benefit of tailoring antiplatelet medications to clopidogrel response in patients undergoing noncoronary endovascular interventions. Further research is needed before a strategy for obtaining and applying clopidogrel responsiveness data can be definitively recommended. Future studies should investigate whether genotyping or PFT is the optimal testing modality, determine whether test result-guided therapy reduces postintervention complications, and evaluate the cost-effectiveness of incorporating clopidogrel response testing in clinical practice.

## Supplementary Material

1

## Figures and Tables

**Fig 1.**
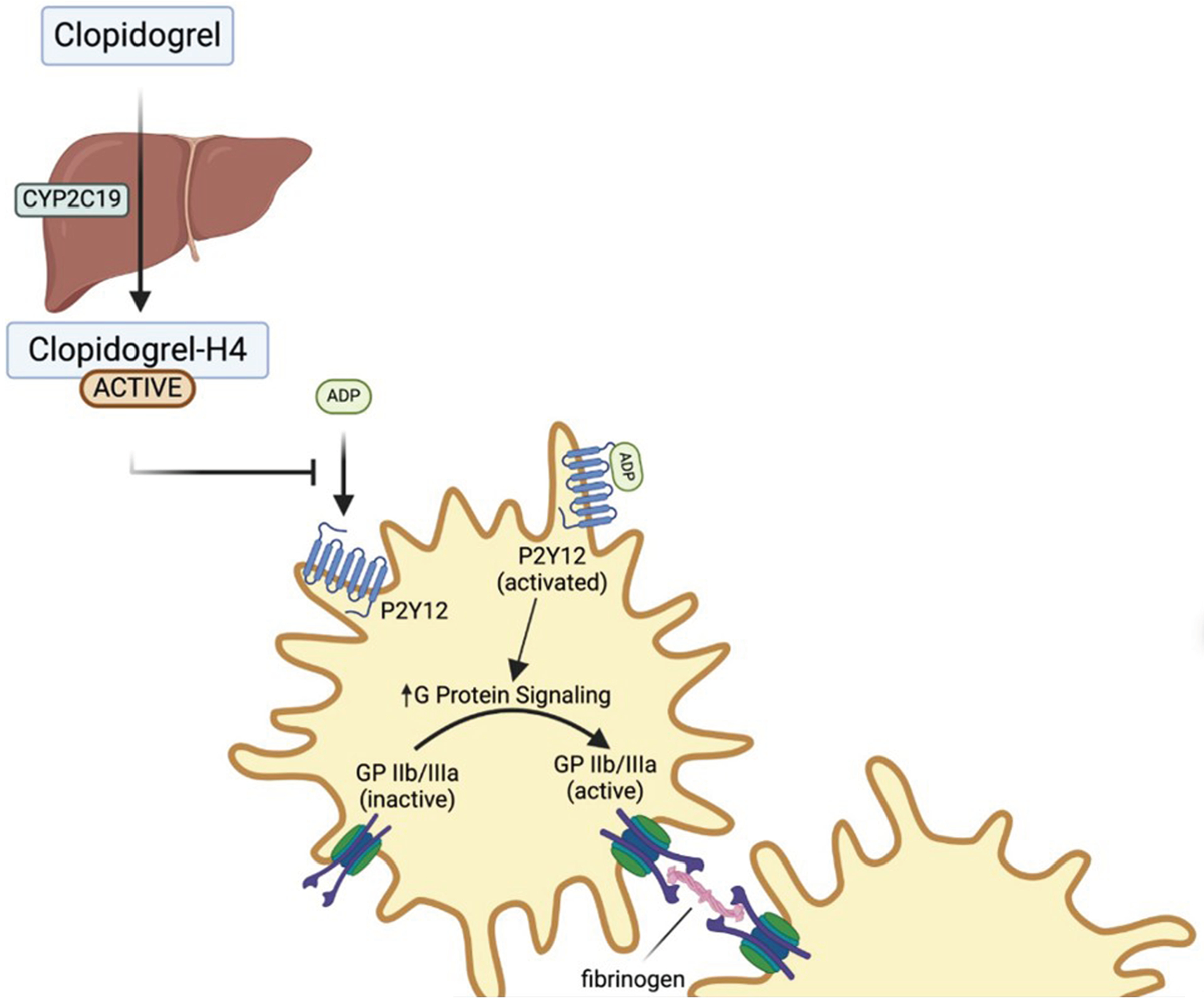
Metabolism of clopidogrel by *CYP450* enzymes. Pathway depicted as described by Brown and Pereira,^17^ Pereira et al,^18^ and Dorsam and Kunapuli.^19^ *ADP*, Adenosine diphosphate. Created with Biorender.com.

**Table.** Predicted phenotype classifications for common *CYP2C19* genotypes

Common genotypes	Predicted phenotype
*1/*17, *17/*17	Ultrarapid metabolizer
*1/*1	Extensive metabolizer
*1/*2, *1/*3, *2/*17, *3/*17	Intermediate metabolizer
*2/*2, *2/*3, *3/*3	Poor metabolizer

Predicted phenotypes as described by González et al.^22^ These are the most common genotypes seen in papers included in the meta-analysis.

## ARTICLE HIGHLIGHTS

- **Type of Research:** Systematic review and meta-analysis
- **Key Findings:** Among patients receiving clopidogrel after endovascular interventions, higher odds of ischemic complications were observed in poor clopidogrel responders identified by genotyping (odds ratio: 2.8, 95% confidence interval: 2.1-3.8, *P* < .001) or by platelet function testing (odds ratio: 6.3, 95% confidence interval: 2.0-20.0, *P* = .02).
- **Take Home Message:** Clopidogrel response impacts clinical outcomes after noncoronary endovascular interventions. However, rigorous studies are needed to establish the best method of evaluating clopidogrel response and determine whether antiplatelet therapy guided by testing for clopidogrel responsiveness reduces periprocedural complications.
