# Supplementary material for: A systematic review and meta-analysis of the impact of clopidogrel responsiveness on ischemic and bleeding complications after noncoronary endovascular procedures
Source: J Vasc Surg. Author manuscript; Available in PMC 2026 Jun 29. (PMC13312069; doi:10.1016/j.jvs.2025.10.101)
Supplement: 1 [file NIHMS2187935-supplement-1.pdf]

Supplementary Table I (online only). *CYP2C19* alleles and their reported frequencies

Allele	Function	Mechanism	Frequency
*1	Wild-type	Functional protein	44.5%-60.5%
*2	Loss of function	Nonfunctional protein	10.1%-34%
*3	Loss of function	Nonfunctional protein	<0.1%-6.7%
*4	Loss of function	Nonfunctional protein	0%-0.2%
*5	Loss of function	Nonfunctional protein	<0.1%
*6	Loss of function	Nonfunctional protein	<0.1%
*7	Loss of function	Nonfunctional protein	<0.1%
*8	Loss of function	Dysfunctional protein	<0.1%
*9	Loss of function	Dysfunctional protein	0%-1.2%
*10	Loss of function	Dysfunctional protein	0%-0.4%
*16	Loss of function	Unknown	<0.1%
*17	Gain of function	Increased transcription	1.5%-29.1%
*22	Unknown	Unknown	0%-0.1%
*24	Unknown	Unknown	<0.1%
*25	Unknown	Unknown	0%
*26	Loss of function	Dysfunctional protein	Unknown
*35	Loss of function	Decreased transcription	Unknown

The effects of each mutation on allele function are reported as described by Brown and Pereira,[17] Ibeanu et al[57] (1999), and Dean and Kane.[37] Allele frequencies differ across ethnicities as reported by Brown and Pereira.[17]

Supplementary Table II (online only). Summary of ischemic complication rates reported in poor clopidogrel responders vs normal responders identified by genotyping

Article	Inclusion criteria	Total subjects, No.	Poor responders, No. (%)	Responders, No. (%)	Outcome measured	Outcomes: Nonresponders with outcome, No. (%)	Outcomes: Responders with outcome, No. (%)	*P* value
Chang et al[28]	Endovascular procedure for CLI Fontaine classifications III or IV	473	223 (47.1)	250 (52.9)	Amputation or revascularization within the study period	92 (41.3)	47 (18.8)	Outcome vs no outcome in nonresponders, $P < .001$; outcome vs no outcome in responders, $P < .001$
Guo et al[29]	Endovascular procedure for TASC II A-C PAD of the SFA	50	26 (52.0)	24 (48.0)	In-stent restenosis on duplex ultrasound imaging or angiography within 1 year	15 (57.7)	4 (16.7)	Outcome in nonresponders vs responders, $P = .004$
Lee et al[30]	Endovascular procedure for CLI Rutherford classifications V or VI	278	125 (45.0)	153 (55.0)	Amputation within 1 year	44 (35.2)	28 (18.3)	Association of nonresponder vs responder status with outcome, $P = .011$
Lin et al[31]	Neuroendovascular procedure for intracranial aneurysms or stenosis	108	31 (28.7)	44 (40.7)	TIA or ischemic stroke within 3 months	3 (9.7)	5 (11.4)	Outcome across all genotype groups[a], $P = .08$

CLI, Critical limb ischemia; *PAD*, peripheral arterial disease; *SFA*, superficial femoral artery; *TASC II*, Trans-Atlantic Inter-Society Consensus for the Management for Peripheral Arterial Disease; *TIA*, transient ischemic attack.
All nonresponders were defined as having ≥1 *CYP2C19*2* or **3* allele on genotyping.
[a]No loss-of-function (LOF), 1 LOF, 2 LOF, or ≥1 gain-of-function allele.

Supplementary Table III (online only). Summary of ischemic complication rates reported in poor clopidogrel responders vs normal responders identified by platelet function testing

Article	Inclusion criteria	Total subjects, No.	Classification of poor responders	Poor responders, No. (%)	Responders, No. (%)	Outcome measured	Outcomes		
							Nonresponders with outcome, No. (%)	Responders with outcome, No. (%)	*P* value
El-Khodary et al[32]	Endovascular (n = 30), open (n = 12), or hybrid procedure (n = 8) for PAD	50	Platelet aggregation ≥60% on platelet aggregation assay	11 (22.0)	39 (78.0)	Major amputation within 1 year	3 (27.3)	1 (2.6)	.029
Muram et al[33]	Transfemoral or transradial revascularization for CAS	100	Addition of ADP resulted in an impedance of >5 Ω on whole-blood impedance aggregometry	15 (15.0)	85 (85.0)	Symptomatic thromboembolic events[a] within 90 days	4 (26.7)	4 (4.7)	.003
Rokosh et al[34]	Transcarotid artery revascularization for CAS	92	PRU ≥194 on VerifyNow P2Y12 assay	30 (32.6)	47 (51.1)	In-hospital ischemic stroke	1 (3.3)	0 (0.0)	.352
						In-hospital MI	0 (0.0)	1 (2.1)	1.00

ADP, Adenosine diphosphate; *CAS,* carotid artery stenosis; *MI,* myocardial infarction; *PAD,* peripheral arterial disease; *PRU,* P2Y12 reaction unit.
All *P* values were determined for outcome in nonresponders vs responders. All platelet function testing was performed after ≥1 week of preoperative clopidogrel.
[a]Any transient or permanent neurological deficit attributable to a thromboembolic event.

Supplementary Table IV (online only). Summary of bleeding complication rates reported in clopidogrel hyper-responders vs responders identified by genotyping or platelet function testing

Article	Inclusion criteria	Total subjects, No.	Hyper-responder classification	Hyper-responders, No. (%)	Responders, No. (%)	Outcome measured	Outcomes: Hyper-responders with outcome, No. (%)	Outcomes: Responders with outcome, No. (%)	Outcomes: *P* value
Kashiwazaki et al[35]	Neuroendovascular procedure for intracranial aneurysms or ICS	66	PFT: P2Y12 percent inhibition ≥74% after ≥14 days of preoperative clopidogrel	15 (22.7)	32 (48.5)	Major bleeding[a] within 30 days	6 (40.0)	2 (6.3)	Outcome in hyper-responders vs responders, *P* = .001
Lin et al[31]	Neuroendovascular procedure for intracranial aneurysms or stenosis	108	Genotyping: CYP2C19*1/*17 or *17/*17	28 (25.9)	44 (40.7)	Intra- or extracranial hemorrhage within 3 months	5 (17.9)	8 (18.2)	Outcome across all genotype groups[b], *P* = .06
Goh et al[36]	Neuroendovascular procedure for intracranial aneurysms or stenosis	47	PFT: P2Y12 percent inhibition ≥72% after ≥3 days of preoperative clopidogrel	7 (14.9)	40 (85.1)	Major bleeding[c] within 6 weeks	3 (42.9)	0 (0.0)	Median P2Y12 percent inhibition among patients with outcome vs without outcome, 94% vs 24%, *P* = .008

ICS, Intracranial carotid artery stenosis; *PFT*, platelet function testing.
[a]Intracranial hemorrhage, retroperitoneal hematoma, large groin hematoma, or bleeding requiring additional intervention.
[b]No loss-of-function (LOF), 1 LOF, 2 LOF, or ≥1 gain-of-function allele.
[c]Intracranial hemorrhage or retroperitoneal hematoma.
